# Novel Insights Into the Multifaceted Functions of RNA n^6^-Methyladenosine Modification in Degenerative Musculoskeletal Diseases

**DOI:** 10.3389/fcell.2021.766020

**Published:** 2021-12-27

**Authors:** Hengzhen Li, WenFeng Xiao, Yuqiong He, Zeqin Wen, Siyuan Cheng, Yi Zhang, Yusheng Li

**Affiliations:** ^1^ Department of Orthopedics, Xiangya Hospital, Central South University, Changsha, China; ^2^ National Clinical Research Center for Geriatric Disorders, Xiangya Hospital, Central South University, Changsha, China; ^3^ Department of Clinical Medicine, Xiangya School of Medicine of Central South University, Changsha, China

**Keywords:** N6-methyladenosine, degenerative musculoskeletal diseases, osteoarthritis, osteoporosis, sarcopenia, degenerative spinal disorders

## Abstract

N^6^-methyladenosine (m^6^A) is an important modification of eukaryotic mRNA. Since the first discovery of the corresponding demethylase and the subsequent identification of m^6^A as a dynamic modification, the function and mechanism of m^6^A in mammalian gene regulation have been extensively investigated. “Writer”, “eraser” and “reader” proteins are key proteins involved in the dynamic regulation of m^6^A modifications, through the anchoring, removal, and interpretation of m^6^A modifications, respectively. Remarkably, such dynamic modifications can regulate the progression of many diseases by affecting RNA splicing, translation, export and degradation. Emerging evidence has identified the relationship between m^6^A modifications and degenerative musculoskeletal diseases, such as osteoarthritis, osteoporosis, sarcopenia and degenerative spinal disorders. Here, we have comprehensively summarized the evidence of the pathogenesis of m^6^A modifications in degenerative musculoskeletal diseases. Moreover, the potential molecular mechanisms, regulatory functions and clinical implications of m^6^A modifications are thoroughly discussed. Our review may provide potential prospects for addressing key issues in further studies.

## Introduction

Emerging evidence has shown that methylation modifications have regulatory effects on the RNA of eukaryotic cells, and the common modifications include N1-methyladenosine (m^1^A), N6-methyladenosine (m^6^A), 5-methylcytosine (m^5^C), 7-methylguanosine (m^7^G), m^1^G, m^2^G, m^6^G, etc. (Shi et al., 2020). m^6^A is the most common of these modifications, accounting for the largest proportion, and approximately 20–40% of all transcripts encoded in mammalian cells are m6A-methylated (Frye et al., 2018). Each mammalian mRNA contains more than three m^6^A sites on average, in the consistent sequence of G (m^6^A) C (70%) and A (m^6^A) C (30%) (Wei et al., 1976; Wei and Moss, 1977). The m^6^A modification was first discovered by Prof. Desrosiers. R and his group in a groundbreaking experiment in the 1970s (Desrosiers et al., 1974). Subsequent studies have shown that it is a dynamic and reversible modification that is widely involved in physiological and pathological processes (Cao et al., 2016), including cellular aging (Casella et al., 2019), cancer progression (Lan et al., 2019) and inflammatory response (Zong et al., 2019). Specifically, m^6^A manipulates the splicing, export, translation and degradation of RNA through methylation and demethylation, controlled by a variety of enzymes, which in turn affect various physiological and pathological processes.

Degenerative musculoskeletal diseases are associated with aging and inflammatory conditions. These diseases include osteoarthritis (OA), osteoporosis (OP), intervertebral disc degeneration disease (IVDD), ossification of the ligamentum flavum (OLF) and sarcopenia (Ikegawa, 2013; Tabebordbar et al., 2013). Currently, a considerable body of epigenetic research is available in this area (Tu et al., 2019; Wijnen and Westendorf, 2019). Alterations in the levels of m^6^A play an important role in the progression of degenerative musculoskeletal diseases (Wu et al., 2018; Liu et al., 2019).

In this review, we present a broad summary of the functions of m^6^A in the development and progression of various degenerative musculoskeletal diseases, with the aim of deepening our understanding of the association between m^6^A and degenerative lesions and exploring the preconceived idea that m^6^A can be a diagnostic marker and therapeutic target for degenerative musculoskeletal diseases in the future.

### RNA m^6^A Modification

**FIGURE 1:**
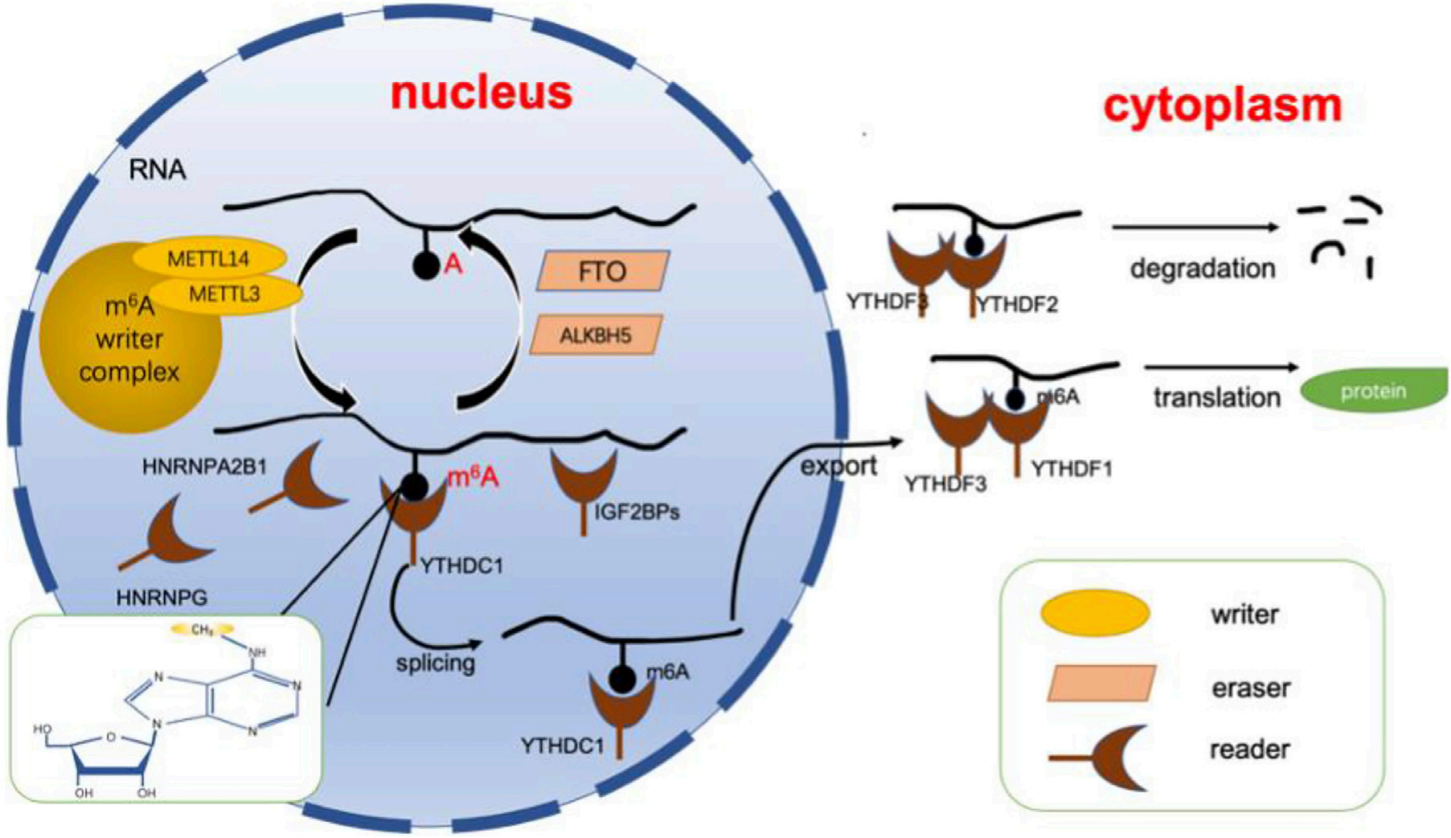
Dynamic regulation of RNA m^6^A modification. The dynamic regulation of RNA m^6^ A modifications relies on writers (including METTL3, METTL14, WTAP, etc.) erasers (including FTO, ALKBH5, etc.), and readers (including YTHDFs, YTHDCs, HNRNPs, etc.). Adenosine located in RNA is recognized by writers for methylation, while erasers can catalyze the demethylation of m^6^A. Finally, the modification is recognized by the reader protein, allowing it to perform its function. ALKBH5, alkB homolog 5; FTO, fat mass and obesity-associated protein. m^6^A, N6-methyladenosine; YTHDF, YTH N6-methyladenosine, RNA binding protein, YTHDC, YTH domain containing protein, HNRNP, heterogenous nuclear ribonucleoprotein.

As mentioned above, the m^6^A modification is a dynamic and reversible epigenetic alteration and controls disease progression by affecting mRNA stability and functionality (Chen et al., 2019a; Li et al., 2020a; Qin et al., 2020). The position of m^6^A in the gene is highly conserved, and it is enriched in the consensus RRACH sequence of stop codons and long internal exons (R = G or A, H = A, C or U) (Dominissini et al., 2012). Current research shows that m^6^A can affect the splicing, translation, export, and degradation of mRNA through three types of key proteins. These three types of proteins are known as m^6^A writers, erasers and readers (Chen et al., 2019b). The writer and eraser proteins dynamically regulate m6A levels, while the readers determine the ultimate fate of mRNA (Shi et al., 2019). In this section, we will analyze and summarize the functions of these three types of proteins (Figure 1).

### m^6^A Writer

m^6^A is incorporated into RNA by a multisubunit writing complex in a highly specific manner (Bokar et al., 1997). This multisubunit writing complex is the m^6^A writer, and the following subunits have been identified: METTL3, METTL14, WTAP, VIRMA, METTl16, etc. METTL3 and METTL14 dominate most of the m^6^A modifications and are the core components of the entire complex. Both of them contain S-adenosylmethionine binding sequences, which can add methyl groups to adenosine and form a heterodimeric complex to regulate m^6^A (Geula et al., 2015; Wang et al., 2016a). Analysis has shown that METTL3 functions as a catalytic subunit, while METTL14 is an important component facilitating binding to RNA (Wang et al., 2016b). WTAP itself does not have methyltransferase activity; it binds to METTL3/14 as a cofactor that helps METTL3/14 localize to nuclear patches and is an essential protein for recruiting substrates (Ping et al., 2014). In addition, it has been shown that WTAP relies on METTL3 to regulate its homeostasis (Sorci et al., 2018). On the other hand, VIRMA functions to promote the binding of m^6^A to the 3′UTR (Yue et al., 2018).

### m^6^A Eraser

In contrast to the function of the m^6^A writer, the m^6^A eraser is responsible for the demethylation of m^6^A to adenosine (Jia et al., 2011). It is important for realization of the dynamic and reversible modification function of m^6^A (Zhao et al., 2017). Demethylation enzymes include fat mass and obesity-associated protein (FTO) and alkB homolog5 (ALKBH5).

The demethylase activity of FTO was first discovered by Prof. He’s group (Jia et al., 2011). It shows homology to the ALKB dioxygenase family. The demethylation function of FTO occurs by oxidizing m^6^A to N6-hydroxymethyladenosine (hm^6^A) and N6-formyladenosine (f^6^A), which eventually becomes simply A (Fu et al., 2013). Although the actual substrate for the action of FTO is N6,2-O-dimethyladenosine (m^6^Am), a modification with a chemical structure identical to that of m^6^A in the base part is found near the 5′ cap in mRNA (Mauer et al., 2017). However, a follow-up study showed that FTO had demethylation activity for both m^6^A and m^6^Am: m^6^A is mainly located in the nucleus, whereas the major substrate in the cytoplasm is m^6^Am (Wei et al., 2018).

ALKBH5 was the second enzyme to be discovered as an m^6^A-based demethylase (Zheng et al., 2013). The role of ALKBH5 can be summarized as follows: 1. Knockdown of the ALKBH5 gene has no effect on the normal growth and development of mice but has an impact on their spermatogenesis. ALKBH5 is enriched in testes and female ovaries, which suggests that the demethylase activity of ALKBH5 is important for germ cell development (Zheng et al., 2013). 2. The altered expression levels of ALKBH5 affect m^6^A modifications, which play an important role in several diseases via the regulation of m^6^A. For example, ALKBH5 expression is decreased in bladder cancer tissues and cells, which correlate with poor patient prognosis. The overexpression of ALKBH5 could inhibit disease progression through the m^6^A-CK2a-mediated glycolytic pathway and increase the sensitivity of bladder cancer to cisplatin (Yu et al., 2021).

### m^6^A Reader

m^6^A readers are a class of proteins that recognize m^6^A modifications on RNA and determine the function of transcripts. These readers include the YT521-B homology (YTH) domain, heterogeneous nuclear ribonucleoproteins, and insulin-like growth factor 2 mRNA-binding proteins.

The crystal structure of the human YTH domain revealed that it contains a recognition pocket consisting of three conserved tryptophan residues for specific recognition of methylation modifications (Luo and Tong, 2014; Xu et al., 2014; Zhu et al., 2014). The most widely studied YT521-B homology (YTH) domains include YTH N6-methyladenosine RNA binding protein 1–3 (YTHDF1-3) and YTH domain containing protein 1–2 (YTHDC1-2). YTH N6-methyladenosine RNA binding protein is mainly localized in the cytoplasm, while YTH domain-containing protein is localized in the nucleus (Reichel et al., 2019). Among them, YTHDF1 promotes the translation of mRNA mainly by affecting the translation mechanism (Wang et al., 2015). On the other hand, YTHDF2 can mediate the degradation of its target m^6^A transcripts by reducing their stability (Li et al., 2018). As a cofactor of YTHDF1 and YTHDF2, YTHDF3 can synergize with both YTHDF1 and YTHDF2 to promote translation and degradation, respectively (Ni et al., 2019). However, YTH domain-containing proteins have other functions. YTHDC1 interacts with m^6^A in nuclear RNA to regulate splicing of premRNA (Kasowitz et al., 2018) and promotes nuclear export of m^6^A-modified RNA (Roundtree, 2017). Interestingly, YTHDC2 seems to be quite important for fertility, as it is mainly enriched in the testis, mediates mRNA stability and translation and regulates spermatogenesis (Hsu et al., 2017). In addition, it promotes the translation of the m^6^A methylation-modified RNA coding region (Mao et al., 2019).

HNRNP is a group of RNA binding proteins responsible for precursor mRNA shearing and stabilization of newly transcribed precursor RNA (Geuens et al., 2016). For instance, hnRNPA2B1 can affect the shear processing of precursor miRNAs by recognizing and binding to sites containing RGm6AC sequences (Alarcón et al., 2015). HNRNPC was one of the first HNRNP proteins identified to be involved in shearing, and it requires oligomerization with other HNRNPC monomers to form a specific binding RNA interaction (Cieniková et al., 2015). HNRNPC preferentially binds single-stranded U-tracts (5 or more contiguous uridines) and affects nascent RNA shearing, translation, etc. (Liu et al., 2015). Finally, HNRNPG contains a low-complexity region that recognizes structural changes mediated by m6A modifications involved in the shearing of cotranscribed precursor mRNAs (Liu et al., 2017; Zhou et al., 2019).

Finally, IGF2BP is able to target transcripts by recognizing GGAC sequences rich in m^6^A modifications; it promotes the translation of mRNA by recruiting mRNA stabilizers such as HuR and MATR3, which enhance the stability of mRNA (Huang et al., 2018).

### Roles of m^6^A in Degenerative Musculoskeletal Disorders

**FIGURE 2:**
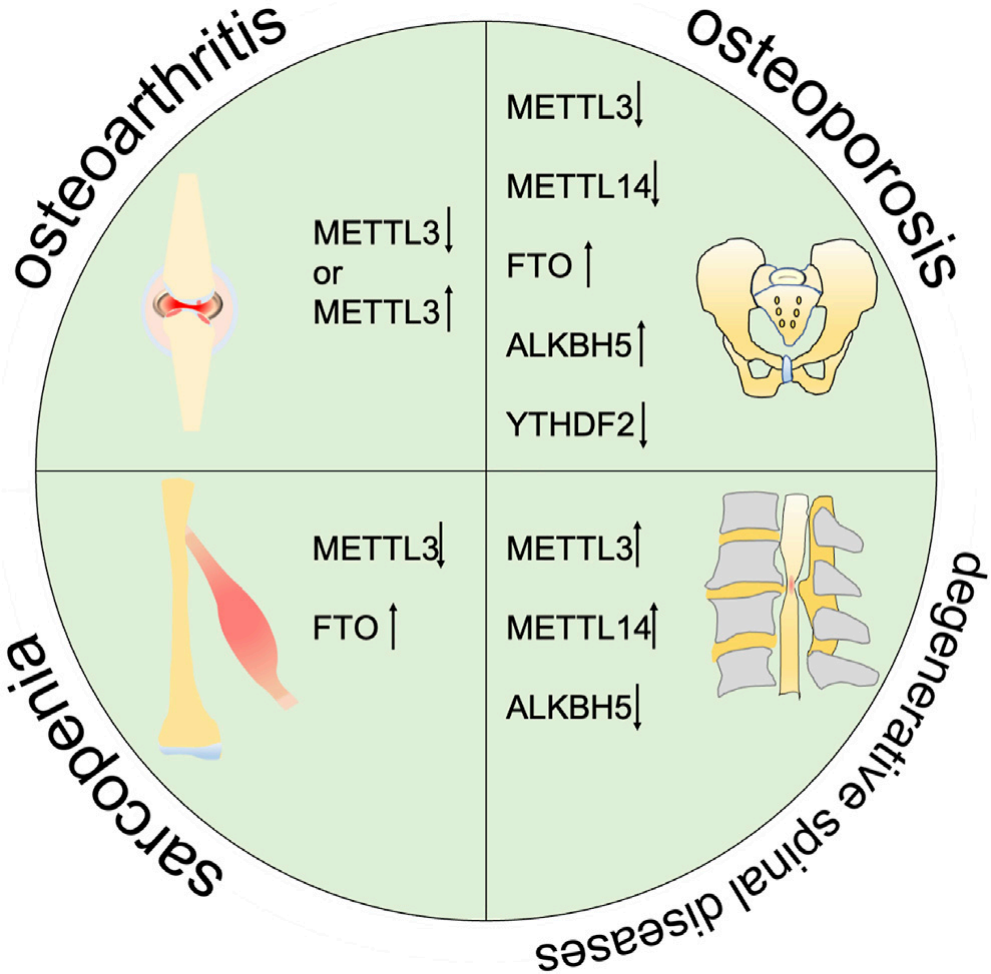
m^6^A is correlated with the progression of multiple degenerative diseases including osteoarthritis, osteoporosis, sarcopenia, and degenarative spinal diseases.

Degenerative musculoskeletal diseases are associated with aging and inflammatory conditions. m^6^A modifications have been considered to be involved in degenerative musculoskeletal diseases. However, the molecular mechanisms and functional details are not fully understood. Thus, we summarize the current evidence on the pleiotropic function of m^6^A in degenerative musculoskeletal diseases (Figure 2, Table 1).

**TABLE 1:** The role of m6A in degenerative musculoskeletal diseases.

disease	m^6^A regulator	Cell type	Target gene/signal pathway	Roles in disease	References
OA	METTL3	ATDC5 Cell	NF- κ B signaling	Promoting inflammatory response, collagen synthesis and degradation, and cell apoptosis in chondrocytes	Liu et al. (2019)
	METTL3	SW1353 cell	NF- κ B signaling	Promoting inflammatory response, degradation of extracellular matrix	Sang et al. (2021)
OP	METTL3	Primary MSCs	PTH/PTH1r signaling	Impairing bone formation	Wu et al. (2018)
	METTL3	BMSCs	PI3K-AKT signaling axis	Inhibiting osteogenic differentiation	Tian et al. (2019)
	METTL3	BMSCs	JAK1/STAT5/C/EBP β signaling axis	Suppressing the early lipid differentiation of BMSCs	Yao et al. (2019)
	METTL3	BMSCs	PremiR-320/RUNX2	Promoting OP development	Yan et al. (2020)
	METTL14	Osteoblasts	miR-103-3p	miR-103-3p can target METTL14 to inhibit osteogenic differentiation	Sun et al. (2021)
	FTO	BMSCs	GDF11- FTO - PPAR γ signal pathway	Promoting differentiation of BMSCs to adipocytes	Shen et al. (2018)
	FTO	BMSCs	miR-149-3p	miR-149-3p promotes osteogenic differentiation by targeting FTO.	Li et al. (2019)
	FTO	BMSCs	miR-22-3p and MYC/PI3K/AKT signal pathway	miR-22-3p in BMSC-derived EVs can inhibit MYC/PI3K/AKT signal pathway by targeting FTO to stimulate osteogenic differentiation	Zhang et al. (2020a)
	ALKBH5	MSCs	PRMT6 mRNA	Inhibiting the osteogenic differentiation of MSCs through PRMT6	Li et al. (2021)
Sarcopenia	METTL3	C2C12 cell	MyoD mRNA	Mettl3 is required for MyoD mRNA expression in proliferative myoblasts	Kudou et al. (2017)
	METTL3	C2C12/MuSCs	-	METTL3 regulates the differentiation of MuSCs	Gheller et al. (2020)
	METTL3	MuSCs	Notch Signaling	Regulating the notch signaling pathway and controlling muscle regeneration and repair with the METTL3-m^6^A-YTHDF1 axis	Liang et al. (2021)
	FTO	C2C12 cell	mTOR-PGC-1α pathway	Regulating mTOR-PGC-1a-mediated intramitochondrial synthesis and muscle cell differentiation	Wang et al. (2017)
	FTO	C2C12 cell	AMPK	Reducing lipid accumulation by inhibiting the demethylase activity of FTO.	Wu et al. (2017)
Degenerative spinal diseases	METTL14	HNPCs	miR-34a-5p	METTL14 promotes he senescence of nucleus pulposus cell by increasing the expression of miR-34a-5p	Zhu et al. (2021)
	METTL3	chondrocytes	PI3K/AKT signaling	METTL3 promotes the degeneration by inhibit the protective effect of PI3K/Akt signaling pathway on endplate cartilage	Xiao et al. (2020)
	METTL3	Primary Ligament Fibroblasts	XIST/miR-302a-3p/USP8 Axis	Regulating the ossification of primary ligament fibroblasts	Yuan et al. (2021)
	ALKBH5	Ligamentum Flavum Cells	AKT pathway	Promoting ligamentum flavum cell osteogenesis by decreasing BMP2 demethylation and activating Akt signaling pathway	Wang et al. (2020b)

ALKBH5, alkB homolog 5; BMSC, bone marrow mesenchymal stem cell; FTO, fat mass and obesity-associated protein; m6A, N6-methyladenosine; METTL3, methyltransferase-like 3; METTL14, methyltransferase-like 14; OA, osteoarthritis; OP, osteoporosis. HNPCs, human nucleus pulposus cell. MuSCs, Muscle-specific adult stem cells.

### m^6^A in Osteoarthritis

Osteoarthritis (OA) is a chronic joint disease represented by symptoms such as pain, stiffness, joint deformity and limited joint movement. Elderly females and overweight people are most affected (Sharma, 2021). Tang. X et al. indicated that the prevalence of knee OA in China was 8.1%, while a later study by Li. Z et al. showed that the prevalence of patellofemoral OA had increased to 23.9% (Tang et al., 2016; Li et al., 2020b). A worldwide study showed that there were approximately 301.1 million prevalent cases of hip and knee OA, which was a 9.3% increase from 1990 to 2017 (Safiri et al., 2020). As the aged population becomes more sophisticated, OA has become one of the most important diseases affecting quality of life, which imposes a huge economic burden on society (Hunter et al., 2014). Pathologically, the main mechanism of OA is the degradation of the articular cartilage matrix, including type II collagen and a small amount of type IX and XI collagen, which ultimately causes total joint damage (Hunter and Bierma-Zeinstra, 2019). In addition, the development of OA is associated with senescent cells, which are linked to aging-related mitochondrial dysfunction and associated oxidative stress (Coryell et al., 2021). Inflammatory factors such as IL-1 β and TNF- α cooperate with chemokines to participate in the progression of OA (Chen et al., 2017)^.^ It is now believed that the study of the relationship between epigenetic regulation and inflammatory factors will be the way forward for OA treatment. Thus, the relationship between m^6^A modifications and OA has attracted the attention of researchers.

Although both Liu. Q et al. and Sang. W et al. concluded that METTL3 affects OA development by regulating the inflammatory response and extracellular matrix (ECM) degradation, and their experiments presented different results. Liu. Q et al. showed that METTL3 expression was increased in IL-1 β -treated ATDC5 cells. Silencing METTL3 expression inhibited the level of inflammatory cytokines and the transactivation of the NF- κ B signaling pathway, which delayed the progression of OA. Moreover, it could inhibit the synthesis of ECM by downregulating the expression of MMP13 and COII-X (Liu et al., 2019). Sang. W et al. showed that METTL3 expression was reduced in patient tissues and in IL-1 β -treated SW1353 cells. Overexpression of METTL3 resulted in decreased levels of inflammatory cytokines and promoted the expression of p-65 protein and *p*-ERK to activate the NF- κ B signaling pathway. Overexpression of METTL3 also regulated the balance between TIMPs and MMPs to affect the degradation of ECM (Sang et al., 2021). The discrepancy in experimental results was speculated to be due to the following two reasons: 1. differences in the selection of cell models: ATDC5 cells and SW1353 cells have a limited ability to mimic primary articular chondrocytes; 2. the normal control selected by Sang. W et al. collected articular cartilage from patients who underwent replacement for femoral neck fractures (for ethical reasons), although whether this is fully consistent with normal human METTL3 expression needs to be reconsidered; 3. Liu. Q et al. verified the expression of METTL3 in experimental osteoarthritis, which might not reflect the actual expression of OP patients. In addition to the methylation enzyme METTL3, the demethylase FTO has also been studied for its effect on the development of OA. It was shown that FTO-mediated overweight could lead to increased susceptibility to OA (arc et al., 2012; Panoutsopoulou et al., 2014). However, both Wang. Y et al. and Dai. J et al. demonstrated that the single nucleotide polymorphism (SNP) rs8044769 of FTO was not associated with OA in the Chinese population, and some other genes may account for it. Therefore, the correlation between FTO and OA needs further investigation (Wang et al., 2016c; Dai et al., 2018).

### m^6^A in Osteoporosis

Osteoporosis (OP), a disease characterized by low bone mass and altered bone microarchitecture (Johnston and Dagar, 2020), is a complex multifactorial disease. Age, sex, BMI (body mass index), postmenopausal women, and previous history of fracture are considered risk factors (Rubin et al., 2013). Altered bone quality and bone microarchitecture in OP cause increased bone brittleness and susceptibility to fracture (Compston et al., 2019), which seriously affect quality of life (Cauley, 2017). Zeng. Q et al. hypothesized that an estimated 10.9 million men and 49.3 million women suffered from OP in China by 2019, and the age-standardized prevalence rates of OP in Chinese men and women over 50 years old were 6.46 and 29.13%, respectively (Zeng et al., 2019). The United States and the United Kingdom spend approximately US$17.9 billion and £4 billion each year on osteoporosis-related fractures (Clynes et al., 2020), which is a huge economic burden for society. However, the current treatment protocols for OP have some issues, such as a long treatment cycle time and poor patient compliance (Qaseem et al., 2017; Estell and Rosen, 2021). Therefore, it is important and intriguing to explore OP treatment from the perspective of epigenetics (de Nigris et al., 2021).

A genome-wide identification study showed that 138, 125 and 993 m^6^A SNPs were associated with density issues of the femoral neck, lumbar spine and heel, respectively, at significant levels (Mo et al., 2018). The differentiation tendency of bone marrow mesenchymal stem cells (BMSCs) is closely associated with the development of OP, and the imbalance between osteogenic and lipogenic differentiation of BMSCs is often considered the basis for the development of OP. BMSC differentiation into adipocytes may lead to decreased bone formation, which contributes to the development of OP (Chen et al., 2016; Qadir et al., 2020). Coincidentally, as an m^6^A-modified demethylase, FTO mediates demethylation to regulate mRNA shearing, which is required for lipogenesis (Zhao et al., 2014). Importantly, Guo. Y et al. found an association between FTO and OP phenotype (Guo et al., 2011). Shen. G et al. found that the GDF11-FTO-PPARγ (peroxisome proliferator-activated receptor γ) axis controls the differentiation of BMSCs to adipocytes and reduces bone formation in OP patients. The main mechanism is that the upregulated GDF11-FTO signaling targets PPAR γ , which is dependent on FTO demethylase activity. This can reduce m^6^A modification of the mRNA encoding PPAR γ , prolong the half-life period, and ultimately contribute to differentiation of BMSCs into adipocytes (Shen et al., 2018). In addition, miR-149-3p can promote the differentiation of BMSCs into osteoblasts by binding to the mRNA 3′UTR of FTO, which in turn inhibits its own expression (Li et al., 2019). Notably, to investigate the effect of extracellular capsule-encapsulated miR-22-3p from bone marrow mesenchymal stem cells on osteogenic differentiation, Zhang. X et al. performed a series of experiments. They found that miR-22-3p in BMSC-derived EVs can inhibit the MYC/PI3K/AKT signaling pathway by targeting FTO to stimulate osteogenic differentiation (Zhang et al., 2020a). Interestingly, although FTO could inhibit the differentiation of BMSCs to osteoblasts in OP, it had a protective effect on differentiated cells. Studies in normal mouse models showed that the demethylase activity of FTO is required for normal bone growth and calcification in mice (Sachse et al., 2018). FTO is also able to avoid genotoxic damage to osteoblasts by stabilizing endoplasmic reticulum stress pathway components, such as Hsp70 (which inhibits NF- κ B signaling pathway activation) (Zhang et al., 2019). As another demethylase, ALKBH5 could also negatively regulate the osteogenic differentiation of MSCs through PRMT6 (protein arginine methyltransferase 6) (Li et al., 2021).

As m^6^A-modified methylesterases, METTL3 and METTL14 have likewise received the attention of researchers. METTL3-and METTL14-mediated m^6^A methylation affects the differentiation of BMSCs through multiple pathways. On the one hand, METTL3 knockdown in mice could decrease the translation efficiency of PTH1r (parathyroid hormone receptor-1) and reduce its expression *in vivo*, which interferes with the osteogenesis of PTH (parathyroid hormone) via the PTH/PTH1r signaling axis to induce an OP-related pathological phenotype (Wu et al., 2018). Moreover, knockdown of METTl3 could inhibit osteogenic differentiation of BMSCs by suppressing VEGF-a expression and activation of the PI3K-AKT signaling pathway *in vivo* (Tian et al., 2019). On the other hand, METTL3 could promote the modification of m^6^A in JAK1 mRNA and reduce JAK1 expression by recognizing and destabilizing JAK1 through YTHDF2, thereby inhibiting the activation of the JAK1/STAT5/C/EBP β signaling pathway. METTL3 could also suppress the early lipid differentiation of BMSCs (Yao et al., 2019). In addition, Yan. G et al. showed that the downregulation of METTL3 in BMSCs could reduce the expression of RUNX2 and PremiR320 by inhibiting their methylation (Yan et al., 2020). RUNX2 is an important regulator of osteogenic precursor cells *in vivo* and is involved in bone mineral deposition and the progression of OP (Komori, 2019). As another m^6^A-modified methylation enzyme, METTL14 can be targeted by miR-103-3p to inhibit osteogenic differentiation. Moreover, it can also modulate miRNA activity through DGCR8 in a feedback-dependent manner, which suggests that the miR-103-3p/METTL14/m^6^A signaling axis is a potential target in the treatment of OP (Sun et al., 2021).

Emerging evidence has shown that the knockdown of the m^6^A-modified reader protein YTHDF2 can enhance the phosphorylation of IKKα/β, IκBα, ERK, p38 and JNK in the NF- κ B and MAPK signaling pathways and then mediate LPS-induced osteoclast formation and inflammation (Fang et al., 2021). This indicates that the role of m6A reader proteins in OP is important, which provides a novel pathway for future research.

In summary, the relationship between m^6^A modifications and OP is closely associated with the regulation of BMSC differentiation. The modalities can be summarized as follows: 1. METTL3 and MEETTL14 can mediate the differentiation of BMSCs toward osteoblasts; 2. FTO can mediate the differentiation of BMSCs toward adipocytes; 3. FTO can protect the cells from genotoxic injury; 4. ALKBH5 negatively regulates the osteogenic differentiation of BMSCs; 5. YTHDF2 reader protein can mediate osteoclast formation. Current research on the relationship between m6A and osteoporosis mainly focuses on the differentiation and regulation of BMSCs. Given that the imbalance of bone remodeling due to abnormal differentiation of osteoclasts is an important pathological basis of osteoporosis and that METTL3 has been shown to regulate osteoclast differentiation (Li et al., 2020c), the mechanism by which m6A modification regulates osteoclast differentiation in osteoporotic patients needs to be further addressed in the future.

Thus, it appears that there may be a dual role of m^6^A modification in the progression of OP. Understanding the mechanism associated with m^6^A modification with this dual relationship could provide promising insight for the prevention and treatment of OP.

### m^6^A in Sarcopenia

Sarcopenia, a disease characterized by a decrease in muscle mass and function associated with age-related progression, was first identified by Rosenberg et al., in 1997 (Rosenberg, 1997). Sarcopenia often results in many adverse outcomes, such as falls, decreased function, fractures and even death. These adverse outcomes can lead to increased hospital stays and exacerbate the sarcopenia process (Coker and Wolfe, 2012; Dhillon and Hasni, 2017; Yeung et al., 2019). The etiology of sarcopenia can be described as follows: 1. Age: muscle content decreases with age and reflects the trend of development. However, the speed of muscle loss in sarcopenia patients is far beyond that in the normal population (Larsson et al., 2019); 2. Chronic low-titer systemic inflammatory state of the body: the body of a sarcopenia patient always presents a chronic low-titer systemic inflammatory state with cachexia, which could increase physical exertion and accelerate muscle decrease (Muscaritoli et al., 2010). Nevertheless, the mechanism of sarcopenia pathogenesis is not yet well understood.

With regard to the relationship between m^6^A modification and sarcopenia, current research has mainly focused on muscle stem cell differentiation. Kudou et al. found that muscle stem cells require MyoD regulators to maintain differentiation potential, and m^6^A modifications of mRNA encoding MyoD are enriched in the 5′UTR. The m^6^A methylation enzyme METTl3 can stabilize MyoD RNA by promoting pro-myogenic differentiation mRNA processing in proliferating cells. Knockdown of METTL3 can significantly downregulate processed MyoD mRNA expression in adult myoblasts (Kudou et al., 2017). Knockdown of METTL3 in mouse C2C12 cells and muscle stem cells can reduce the level of m^6^A modification and lead to premature differentiation of adult myoblasts, suggesting an important role of METTL3 in m^6^A regulation (Gheller et al., 2020). METTL3 can enhance protein expression by increasing mRNA m^6^A modification via the Notch signaling pathway and increase the translation efficiency of mRNAs through the YTHDF1 reader protein. This suggests that METTL3 is essential for regulating muscle stem cells and promoting muscle injury recovery (Liang et al., 2021).

Similarly, FTO demethylases have also been found to be involved in the regulation of muscle stem cells. Increased expression of FTO is observed during muscle cell differentiation and regulates mTOR-PGC-1a-mediated intramitochondrial synthesis through its own demethylase activity (affecting muscle cell differentiation) (Wang et al., 2017). In addition, the expression of AMPK (AMP-activated protein kinases) is a key regulator of skeletal muscle lipid metabolism and m^6^A modification in skeletal muscle. These proteins showed a negative correlation with lipid accumulation in skeletal muscle. Lipid accumulation may be reduced by inhibiting the demethylase activity of FTO and increasing the level of m^6^A modification (Wu et al., 2017).

In summary, although the existing evidence does not directly verify the relationship between m^6^A modification and sarcopenia, the ability of m^6^A to regulate the differentiation of muscle stem cells will provide us with a future direction. Given the variety of sarcopenia mouse models that have been established (Xie et al., 2021), novel methods of sarcopenia research can be developed. Interestingly, given the regulatory role of FTO in muscle differentiation and lipid accumulation in skeletal muscle, FTO may be considered a key regulatory factor specifically in sarcopenic obesity (high-risk disease characterized by both sarcopenia and obesity (Batsis and Villareal, 2018)).

### m^6^A in Degenerative Spinal Disease

Degenerative spinal disorders are a group of age- and aging-related structural abnormalities of the spine, including cervical spondylosis, lumbar disc herniation, spinal stenosis and posterior longitudinal ligament calcification (Ailon et al., 2015; Davies et al., 2018). These constitute a type of clinical syndrome caused by degenerative alternations or long-term strain as age increases. A structural imbalance in the spine initiates repair in the body and stimulates bone hyperplasia, ligament thickening and ossification, which eventually lead to the emergence of spinal cord, nerve root or vertebral dynamic compression. This imbalance can seriously affect the quality of life of patients and even endanger life (Wang et al., 2016d; Badhiwala et al., 2020). Abnormal nucleus pulposus cells are a crucial cause of lower back pain (a common chronic inflammatory pain closely related to disc degeneration in which IL-1 and TNF- α are key factors (Cunha et al., 2018; Wang et al., 2020a)). Zhu. H et al. showed that TNF- α and TNF- α can promote the expression of miR-34a-5p through the methylation enzyme activity of METTL14 in myeloid cells, which may increase the m^6^A modification of the mRNA encoding miR-34a-5p (targeting the utility of SIRT1 inhibition). Eventually, this promotes the senescence of nucleus pulposus cells (Zhu et al., 2021). As another methylesterase, METTL3 is able to promote inflammation by binding DGCR8 to positively regulate the m^6^A modification level of pri-miR-365-3p in a CFA-induced chronic inflammation model (Zhang et al., 2020b). In IVDD, degeneration of endplate chondrocytes may also lead to pathological alterations. Xiao. L et al. found that METTL3-mediated m^6^A modification was closely associated with degeneration (Xiao et al., 2020). METTL3 expression was upregulated in IL-1 β -mediated inflammatory cells: METTL3 upregulation promoted the breakdown of pri-miR-126-5p to increase miR-126-5p expression. Subsequently, miR-126 could downregulate PIK3R2 expression to inhibit the protective effect of the PI3K/Akt signaling pathway (Xiao et al., 2020). METTl3 increases the level of m6A modification of lncRNA XIST during posterior longitudinal ligament ossification and subsequently affects the ossification of primary ligament fibroblasts by influencing the miR-302a-3p/USP8 axis (Yuan et al., 2021). During ligamentum flavum ossification, the ALKBH5 demethylase can promote ligamentum flavum cell osteogenesis by decreasing BMP2 demethylation and activating the Akt signaling pathway (Wang et al., 2020b).

Thus, although research on the role of m^6^A in the process of spinal degeneration is still in its infancy, a close association between the regulation of m^6^A modifications and spinal degeneration has been identified. Both the METTL3 and METTL14 methylation enzymes and the ALKBH5 demethylase can influence the progression of spinal degeneration by regulating the level of m^6^A modifications (affecting the level of inflammation or differentiation tendency). The excellent studies described here provide novel insight for the diagnosis and treatment of degenerative spinal disorders in the future.

### Perspective

Currently, accurately describing the specific mechanisms of m^6^A in degenerative musculoskeletal diseases remains a great challenge. The impact of m^6^A modifications on degenerative musculoskeletal diseases remains to be addressed. First, the current SNP detection methods, such as high-resolution and high-throughput detection, need to be improved. Second, research on OA, sarcopenia and degenerative spinal diseases is relatively limited, and we hope that subsequent investigators will more thoroughly examine the mechanisms involved. Third, although an important role of YTHDF2 in degenerative musculoskeletal diseases has been observed, the role of the reader protein has been less well investigated (Fang et al., 2021). Finally, current evidence suggests that targeting m^6^A modifications may be a promising therapeutic option (Peng et al., 2019; Bedi et al., 2020). However, more in-depth studies on safety and efficacy are still needed.

## Conclusion

Recently, researchers have begun to investigate the role and importance of m^6^A modifications in a variety of diseases. However, only a small number of these studies have focused on degenerative issues. In this review, we summarize the role and regulatory mechanisms of m^6^A in the pathogenesis of degenerative musculoskeletal diseases. During transcription, the level of transcript m^6^A modification is closely associated with the development and repair of bones, muscles and soft tissues. The regulation of the m^6^A modification level at the lesion site requires functional coordination among writer, eraser and reader proteins, and the abnormal expression of each of these proteins may contribute to exacerbating degeneration. Therefore, the dynamic balance of m^6^A modifications is crucial for degenerative musculoskeletal diseases. Unfortunately, the current treatment options for degenerative musculoskeletal diseases are not yet well understood, and most patients are ultimately likely to receive surgical treatment. Research on the relationship between m^6^A modifications and degenerative musculoskeletal diseases will provide us with novel insights for the diagnosis and treatment of these diseases to control their progression and long-term prognosis by regulating m^6^A modification.
